# The rat intervertebral disk degeneration pain model: relationships between biological and structural alterations and pain

**DOI:** 10.1186/ar3485

**Published:** 2011-10-13

**Authors:** Jae-Sung Kim, Jeffrey S Kroin, Xin Li, Howard S An, Asokumar Buvanendran, Dongyao Yan, Kenneth J Tuman, Andre J van Wijnen, Di Chen, Hee-Jeong Im

**Affiliations:** 1 Department of Biochemistry, Rush University Medical Center, Cohn Research BD 516, 1735 West Harrison Street, Chicago, IL 60612, USA; 2 Departement of Anesthesiology, Rush University Medical Center, 1735 West Harrison Street, Chicago, IL 60612, USA; 3 Department of Orthopedic Surgery, Rush University Medical Center, 1735 West Harrison Street, Chicago, IL 60612, USA; 4 Department of Cell Biology, University of Massachusetts, 55 Lake Avenue, North Worcester, MA 01655-0106, USA; 5 Department of Internal Medicine, Section of Rheumatology, Rush University Medical Center, 1735 West Harrison Street, Chicago, IL 60612, USA; 6 Department of Bioengineering, University of Illinois at Chicago, 851 South Morgan Street, 218 SEO, Chicago, IL 60607-7052, USA

**Keywords:** lumbar disk degeneration, pain pathway, chronic back pain, animal model, drug test, pain intervention

## Abstract

**Introduction:**

Degeneration of the interverterbral disk is as a cause of low-back pain is increasing. To gain insight into relationships between biological processes, structural alterations and behavioral pain, we created an animal model in rats.

**Methods:**

Disk degeneration was induced by removal of the nucleus pulposus (NP) from the lumbar disks (L4/L5 and L5/L6) of Sprague Dawley rats using a 0.5-mm-diameter microsurgical drill. The degree of primary hyperalgesia was assessed by using an algometer to measure pain upon external pressure on injured lumbar disks. Biochemical and histological assessments and radiographs of injured disks were used for evaluation. We investigated therapeutic modulation of chronic pain by administering pharmaceutical drugs in this animal model.

**Results:**

After removal of the NP, pressure hyperalgesia developed over the lower back. Nine weeks after surgery we observed damaged or degenerated disks with proteoglycan loss and narrowing of disk height. These biological and structural changes in disks were closely related to the sustained pain hyperalgesia. A high dose of morphine (6.7 mg/kg) resulted in effective pain relief. However, high doses of pregabalin (20 mg/kg), a drug that has been used for treatment of chronic neuropathic pain, as well as the anti-inflammatory drugs celecoxib (50 mg/kg; a selective inhibitor of cyclooxygenase 2 (COX-2)) and ketorolac (20 mg/kg; an inhibitor of COX-1 and COX-2), did not have significant antihyperalgesic effects in our disk injury animal model.

**Conclusions:**

Although similarities in gene expression profiles suggest potential overlap in chronic pain pathways linked to disk injury or neuropathy, drug-testing results suggest that pain pathways linked to these two chronic pain conditions are mechanistically distinct. Our findings provide a foundation for future research on new therapeutic interventions that can lead to improvements in the treatment of patients with back pain due to disk degeneration.

## Introduction

The intervertebral disk has a unique structure composed of a tough outer ring, the annulus fibrosus (AF), as well as a gelatinous inner core, the nucleus pulposus (NP). Although there are many causes of low-back pain, symptomatic degeneration of the intervertebral disk is thought to be the leading cause of chronic discogenic pain syndrome in the Western world [1]. Despite extensive study of the degenerative process in the intervertebral disk, the exact mechanism of discogenic back pain has not been elucidated. Many features of discogenic pain have not yet been explained, including the fact that only a minority of patients with severe degenerative changes of the disk are clinically affected by severe, chronic back pain. Clearly, more information regarding the pathogenesis of discogenic pain is needed before a rational biological or pharmacological treatment strategy for this pervasive disease process can be designed.

Animal pain models are essential for understanding the complexities of pain and the development and testing of new therapies. Joint pain is clinically assessed by observing human movement and reflexes to touch, as well as by asking patients to report the quality and intensity of their pain. Pain in animals can be measured by observing (1) pain-related behaviors, such as vocalization of pain or biting, licking or shaking of the affected limb; and/or (2) responses to thermal or mechanical stimuli. The rabbit disk puncture model has been beneficial in the study of biological mechanisms of disk degeneration and in testing therapeutics for disk regeneration. After annulus needle puncture, the rabbit disk slowly and progressively degenerates [2]. The degeneration can be quantitatively assessed by conventional radiography, magnetic resonance imaging (MRI), and histological examination. Nevertheless, rabbits tend to show minimal pain behavior during disk degeneration in this model. Typically, when rabbits sense pain, they limit their activity and fail to thrive. Rabbits do not display assessable pain behavior; therefore, they are not suitable for the study of discogenic back pain.

Because of rats' sensitive behavioral responses (for example, vocalization), rat models have been used extensively to study chronic inflammatory and neuropathic pain in hind limbs and to evaluate the pharmacokinetics of analgesics [3-5]. Most studies of disk degeneration have been performed in the rat tail rather than in lumbar disks [6,7], in part because of its anatomical accessibility and minimal surgical morbidity. Intervertebral disk damage in the rat tail may provoke a painful response. However, unlike spine disks, tail disks are not weight-bearing, and it is unclear anatomically which neural structures or components are involved. Thus, it would be rather difficult to interpret the resulting nociceptive pathways. A recent study by Olmarker [8] indicates that a rat model with measurable pain behavior may be suitable for studying discogenic back pain. They reported that disk puncture with a needle (0.4 mm in diameter) in rats induced measurable pain behavior with increased grooming and whole-body ("wet dog") shaking.

In our present study, we sought to establish novel correlations between pain and pathological changes in the disk structure. We developed an animal model for chronic discogenic back pain that is amenable to assessment of behavioral hyperalgesia. We examined biological links between cellular and structural alterations within disk components and the development of symptomatic chronic back pain. We also characterized pain modulators associated with symptomatic back pain in the dorsal root ganglion (DRG) and the spinal cord. Furthermore, we performed pharmacological tests to explore potential therapeutic analgesic modulation of chronic back pain.

## Materials and methods

### Induction of disk degeneration

This study was approved by the Institutional Animal Care and Use Committee of Rush University Medical Center. Adult Sprague Dawley rats (250 to 300 g) were fasted for 24 hours prior to surgery to allow easier access to the ventral spine. The animals were anesthetized with 1.5% isoflurane in oxygen (1 L/minute), which was maintained via a mask. The surgery was performed using aseptic techniques. With the animal in the supine position, the abdominal hair was shaved, scrubbed with a topical antiseptic solution (chlorhexidine gluconate) and alcohol three times, and the skin over the lower abdomen was opened with a #15 scalpel blade. A midline ventral abdominal incision of 1.5 to 2 cm was made, and the abdominal viscera were gently retracted to allow visualization and access to the spine and lumbar disk space. The viscera were irrigated with prewarmed sterile saline solution and covered with moist, sterile gauze to maintain tissue hydration.

To induce disk degeneration, disks were punctured with a microsurgical drill (0.5 mm in diameter and 0.25 mm^2 ^area, equivalent to a 25-gauge needle) inserted into the intervertebral disks (L4/L5 and L5/L6) to a depth of 2 mm. This was accomplished by using a polyethylene stopper sleeve (BD Intramedic™ Polyethylene Tubing (Non-Sterile) (PE 50), catalog no. 427411; BD, Franklin Lakes, NJ, USA) cut 2 mm shorter than the length of the drill. Muscles were closed with 3-0 silk suture, and all skin margins were closed with 4-0 nylon suture. The animals were then taken off anesthesia and kept warm in a clean cage postsurgery. For two days following surgery, all animals were injected intramuscularly with buprenorphine 0.1 mg/kg twice daily as a postsurgical analgesic. The animals were then monitored to be certain that there were no serious impairments due to the surgery. Another group of animals had their disks punctured with a larger drill (0.8 mm in diameter and 0.64 mm^2 ^area, equivalent to a 21-gauge needle). For the sham surgery control group, disks were exposed in the same way as in the experimental group, but with no puncture (*n *= 12 in each animal group). Degeneration of disks was assessed on the basis of the biochemical, histological and imaging analyses described in the sections that follow. A neuropathic pain animal model was generated by using a L5 spinal nerve ligation model [9] for comparison with tissue protein and RNA levels in the disk degeneration model.

### Animal behavioral tests

#### Vocalization threshold in applied pressure test

The vocalization threshold based on the force of an applied force gauge (SMALGO algometer; Bioseb, Vitrolles, 13845 France) was measured by pressing the 0.5-cm^2 ^device tip directly on the dorsal skin over the punctured disks (L4/L5) and comparing this threshold value with the force threshold applied to the disks (L4/L5) in the sham surgery control group. The force was slowly increased 100 *g*/second until an audible vocalization was heard. A cutoff force of 1,000 *g *was used to prevent tissue trauma. The tests were carried out in duplicate, and the mean value was taken as the nociceptive threshold. Postoperative testing was delayed until one week after surgery to allow the abdominal tissue to heal. This pain test is clinically relevant, as patients' pain is tested by mechanical pressure on disks to assess their degree of back pain [10-12].

#### Mechanical allodynia

After allowing the rats to become acclimated to a wire mesh grid covered with a clear plastic cage for 15 minutes, a calibrated set of von Frey filaments (Stoelting, Wood Dale, IL, USA) was applied from below to the plantar hind paw to determine the 50% force withdrawal threshold [13]. The filament forces ranged from 0.04 to 15 *g*, beginning with 2.0 *g*. The filament was applied to the skin with enough pressure to buckle and was maintained for up to six seconds. A brisk lifting of the foot was recorded as a positive response. Mechanical allodynia thresholds in spondylosis models were measured for both legs as indicators of secondary hypersensitivity. The average of the left and right sides was calculated on the basis of data derived from all leg pain tests [14].

#### Motor incoordination

Rats were placed on a rotating rod (the rotarod performance test) apparatus at 10 rpm, and the latency to fall time was recorded for coordination and balance capability. The maximum cutoff latency was 300 seconds.

### Drug treatment

For drug administration experiments beginning at seven weeks after induction of disk degeneration, the rats were gently restrained and given 0.5 ml of drug solution either intraperitoneally (i.p.) or by oral gavage (p.o.). The drugs tested were a α_2_δ_1 _subunit calcium channel blocker, pregabalin 20 mg/kg p.o. (Pfizer, Piscataway, NJ, USA); a mixed cyclooxygenase (COX-1 and COX-2) inhibitor, ketorolac tromethamine 20 mg/kg i.p. (Sigma-Aldrich, St Louis, MO, USA); a COX-2 selective inhibitor, celecoxib 50 mg/kg p.o. (Pfizer); a μ-opioid agonist, morphine sulfate 6.7 mg/kg i.p.; and drug vehicle. Each drug administration was performed at three- to four-day intervals in the same rats to minimize the number of rats used while avoiding residual drug effects of prior treatments. Pressure hyperalgesia was performed at a maximum of four time points after drug injection to limit local tissue hypersensitivity due to the repeated noxious stimulus itself. The drug vehicle for i.p. delivery was saline (0.9% sodium chloride injection), and the drug-suspending vehicle ORA-PLUS (Paddock Laboratories, Inc, Minneapolis, MN, USA) was used for p.o. administration. None of drug doses used in this study caused sedation or motor incoordination in naïve rats. Sedation in naïve rats was evaluated by measuring spontaneous exploratory activity (ambulation and rearing) with a photobeam activity monitor as previously described [15]. Briefly, photobeam-generated counts were recorded during 30 minutes of activity 1 hour after injection. The morphine injection (6.7 mg/kg) (*n *= 8) versus saline injection (*n *= 8) ambulation counts (means ± SEM) were saline 566 ± 57 and morphine 571 ± 95 (*P *= 0.961). The rearing ambulation counts were saline 127 ± 13 and morphine 111 ± 20 (*P *= 0.505). Our data suggest no statistical difference in spontaneous exploratory activity associated with 6.7 mg/kg morphine.

### Histological and data analyses

**Figure 1:**
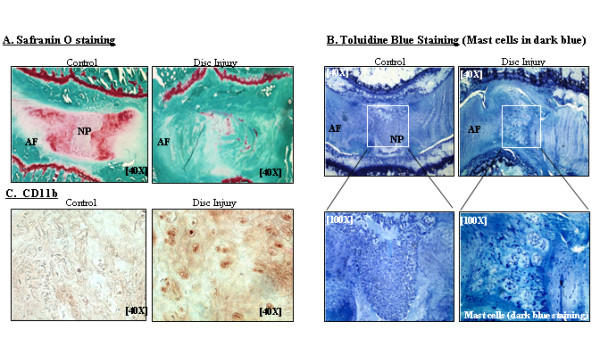
**Axial sections of the L4/L5 lumbar disks showing immunohistochemical staining with Safranin O, toluidine blue (nine weeks after surgery) and CD11b**. **(A) **Safranin O-stained sections of sham control (left) and disk injury (right). **(B) **Toluidine blue-stained sections of sham control (top left) and traumatic disk puncture (top right) (original magnification, ×40). Lower panels show enlargements (original magnification, ×100) of boxed portions of top left and top right panels. Mast cells are indicated by dark blue staining. **(C) **CD11b-stained sections (original magnification, ×40). Immune cells are shown in dark blue staining. AF = annulus fibrosus; NP = nucleus pulposus.

The animals were killed, and each vertebral lumbar motion segment was dissected aseptically. The tissues were fixed in 4% paraformaldehyde and decalcified in ethylenediaminetetraacetic acid solution (changed every five days). The decalcified disk was cut in the transverse plane and embedded in paraffin. Serial frontal disk sections of exactly 5-μm thickness were obtained to prepare slides. Safranin O Fast Green staining was performed to assess general morphology and loss of proteoglycan in ground cartilage substance. We compared all harvested disks using the grading scale published by Masuda *et al*. [16]. On the Masuda scale, point scores range from 4 to 12, where normal (Figure 1A (left panel): Control) is 1 point for each of the following 4 categories (total of 4 points = grade 4): category I = AF, category II = border between the AF and NP, category III = cellularity at the NP and category IV = matrix of the NP. Because each category has a maximum of 3 points, a total score of 12 points (grade 12) represents severe degeneration (Figure 1A (right panel): Disk Injury). An unblinded investigator grouped the slides by animal. These groups were then randomized for grading. The grading was repeated by an orthopedic histologist who was blinded to the animal treatments. The two independent examinations were performed, and the repeatability of grading on the two occasions was determined using Cohen's κ statistic using an internet-based program [17]. *P *< 0.05 was defined as significant for all tests [16,18].

Toluidine blue staining was carried out to assess the general morphology of mast cells (which appear dark blue) [19]. Immunohistochemistry was performed using anti-CD11b antibody according to established methods [20]. In brief, after blocking endogenous peroxidases, tissue sections were incubated overnight with primary rat anti-mouse CD11b antibody (Cell Signaling Technology, Danvers, MA, USA) for macrophage binding. The tissue sections were then incubated with a secondary biotinylated antibody, followed by incubation with a streptavidin-horseradish peroxidase conjugate and development with 0.5 mg/ml diaminobenzidine.

### Imaging analyses for intervertebral disk height index

Disk heights were determined using the MX-20 Specimen Radiography System (Faxitron Bioptics LLC, Lincolnshire, IL, USA). At the end of the experiment period, excised lumbar spinal motion segments (L4/L5 and L5/L6) were radiographed by placing the spine segments directly on the X-ray film for five minutes with the energy for 10 kV. The image was digitized, and the gross disk heights were evaluated and graded.

**Figure 2:**
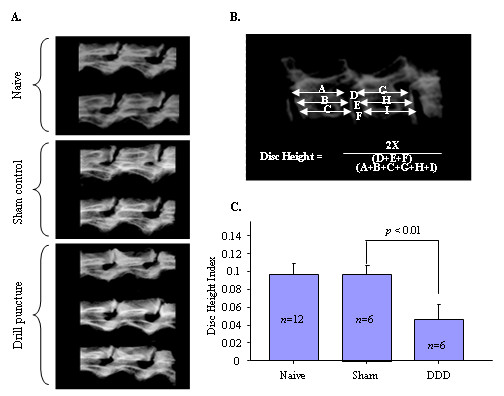
**Using digitized X-ray images, we analyzed measurements, including the vertebral body height and intervertebral disk height, with imaging software**. **(A) **Digitized X-ray images of intervertebral disk segments obtained to compare presurgery (naïve), sham control and drill puncture. **(B) **Intervertebral disk height was calculated by averaging the measurements obtained from the anterior, middle and posterior portions of the intervertebral disk and dividing that average by the average adjacent vertebral body height. **(C) **The disk height index was measured nine weeks after surgery. The disk height index of disk degeneration (DDD) was significantly decreased compared to sham control (*P *< 0.01).

Two examiners who were blinded to the treatment independently conducted measurements of disk height. Intervertebral disk height was calculated by using modified versions of previously established methods [16]. The intervertebral disk height index was obtained by averaging measurements obtained from anterior, middle and posterior portions of the intervertebral disk and dividing the measurements by the average of adjacent vertebral body heights (see Figures 2A and 2B). To evaluate changes in the intervertebral disk height of degenerated disks, those disk heights were normalized to the measured sham disk heights (disk height index = degenerative disk height (*n *= 6)/sham control disk height (*n *= 6).

### Quantitative real-time polymerase chain reaction

Rats with removed NPs or sham controls were killed at week 9. Bilateral lumbar DRGs and spinal cord levels L4/L5 and L5/L6 were harvested, and relative mRNA levels of pain-related genes were analyzed by quantitative real-time PCR (qRT-PCR). Total RNA was isolated using the TRIzol reagent (Invitrogen, Carlsbad, CA, USA) according to the instructions provided by the manufacturer. Reverse transcription (RT) was carried out with 1 μg of total RNA using the ThermoScript™ RT-PCR System (Invitrogen) for first-strand cDNA synthesis. For RT-PCR, cDNA was amplified using the MyiQ2 Two-Color Real-Time PCR Detection System (Bio-Rad Laboratories, Hercules, CA, USA). A threshold cycle (Ct) value was obtained from each amplification curve by using iQ5 Optical System Software provided by the manufacturer (Bio-Rad Laboratories). Relative mRNA expression was determined using the ^ΔΔ^Ct method as detailed by the manufacturer (Bio-Rad Laboratories). The primers employed were (forward) 5'-TCTGTGCCTCAGCCTCTTCTCATT-3', (reverse) 5'-TTGGGAACTTCTCCTC CTTGT TGG-3' for TNF-α (National Center for Biotechnology Information (NCBI) accession NM_012675.3); (forward) 5'-GAAGCTGTAGTATTTGTCACCAAGC-3' and (reverse) 5'-CTAATGTACTTCTGGAC CCATTCCT-3' for monocyte chemoattractant protein 1 (MCP-1) (NCBI M_57441.1); (forward) 5'-TCTGACAAACCTAGAACATGTGGA-3' and (reverse) 5'-TCACGTAGA AACTGTAAGTCTTTGACA-3' for Toll-like receptor 4 (TLR4) (NCBI accession NM_019178.1); 5'-AATGAGAGTGAGTCAGGCAGCCAA-3' and (reverse) 5'-ATGGACC GCTGCATGTTGATAGG A-3' for α_2_δ_1 _(NCBI accession NM_012919.2); (forward) 5'- AGGGCAGTTGGACAGTCATTGGTA-3' and (reverse) 5'- TTCAACTCTCATCCACCTT GGCGA-3' for brain-derived neurotrophic factor (BDNF) (NCBI accession NM_012513); (forward) 5'-TCTAGTGTCACTGCCCAGAAGAGA-3' and 5'-GGCACAAAGTTG TCCTTCACCACA-3' for calcitonin gene-related protein (CGRP) (NCBI accession NM_001033956.1); (forward) 5'-TTCCCACCACTGCTCAAGATG-3' and (reverse) 5'-TGGCTGACAGGGTTGCAA-3' for galanin (NCBI accession NM_033237.1); (forward) 5'-AAGCTTAAGTGGGACAACCAGAAA-3' and (reverse) 5'-GTTCTCCTGGGACCGAGTCA-3' for dynorphin (NCBI accession NM_01937 4.3); (forward) 5'-AGATCCAGCCCTG AGACACTGATT-3' and (reverse) 5'-TGGAAGGGTCTTCAAGCCTTGTTC-3' for neuropeptide Y (NPY) (NCBI accession NM_012614.1); (forward) 5'-TGGGCAACGTAGTGGTGATA-3' and (reverse) 5'-CACGGCTGTCATGGAGTAGA-3' for neurokinin 1 (NK-1) (NCBI accession NM_012667.2); (forward) 5'-TGGTCAGAT CTCTCACAAAAGG-3' and (reverse) 5'-TGCATTGCGCTTCTTTCATA-3' for substance P (NCBI accession NM_001124768.1); (forward) 5'-TGTCACCAACTGGGACGATATGGA-3' and (reverse) 5'-AGCACAGGGTGCTCCTCA-3' for β-actin (NCBI accession NM_031144). The deviations in samples represent three different donors in three separate experiments.

### Protein extraction and Western blot analysis

Tissue lysates obtained from rat were prepared using modified radioimmunoprecipitation assay buffer as previously described. Total protein concentrations of tissue lysates were determined by using bicinchoninic acid protein assays (Pierce Biotechnology, Rockford, IL, USA). Equal amounts of protein were resolved by 10% SDS-PAGE and transferred onto nitrocellulose membrane for Western blot analyses as described previously. Immunoreactivity was visualized using the Amersham ECL Western Blotting System (GE Healthcare Bio-Sciences Corp, Piscataway, NJ, USA).

### Statistical analysis

The significance of differences among means of data derived by radiograph measurements was analyzed by analysis of variance for repeated measurements, and Fisher's protected least significant difference test was used as a *post hoc *test. All data are expressed as means ± SEM. Statistical analysis was performed using the StatView version 5.0 software program package (SPSS, Inc, Chicago, IL, USA). The Cohen's κ value was calculated by using an internet-based program [17]. *P *< 0.05 was defined as statistically significant for all tests. Algometer pressure threshold measurements and von Frey filament threshold determinations over the seven-week postoperative period between the disk injury group and sham control group were compared using a mixed general linear model with repeated measures (SAS statistical software; SAS Institute Inc, Cary, NC, USA). A step-down Bonferroni correction was used for multiple comparisons. Pressure pain measures after drug injection were compared among different drugs using a mixed general linear model with repeated measures. For the straight leg-raising test, vocalizations were compared over the seven-week testing period using the Friedman test with a *post hoc *Wilcoxon signed-rank test. Measures were compared between experimental animals and control animals with the Mann-Whitney *U *test or the independent samples *t*-test.

## Results

### Disk puncture causes symptomatic chronic back pain

**Figure 3:**
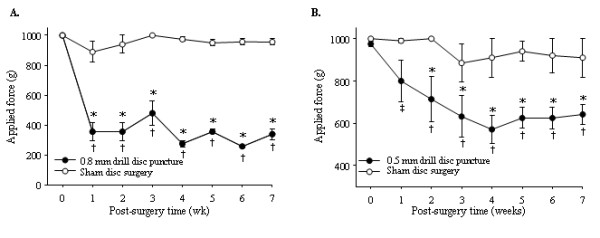
**Behavioral pain assessments over a seven-week time period**. Pressure hypersensitivity of the lower back was determined by measurement of the vocalization threshold to an applied force gauge (algometer). **(A) **Graph showing data derived from animals that underwent traumatic puncture by a drill (0.8 mm in diameter and 0.64 mm^2 ^area) (*n *= 12) compared with the sham surgery group (*n *= 12). *P < 0.001 versus sham group; †*P *< 0.001 versus presurgery baseline (time 0). **(B) **Graph showing data derived from animals punctured with a smaller drill (0.5 mm in diameter and 0.25 mm^2 ^area) (*n *= 6) were compared with the sham surgery group (*n *= 6). *P < 0.05 versus sham group; †*P *< 0.001 versus presurgery baseline; ‡*P *< 0.005 versus presurgery baseline.

Our previous attempts using a 21-gauge drill (0.8 mm diameter and 0.64 mm^2 ^area) failed to induce gradual back pain but instead generated rather traumatic, rapid development of pressure hyperalgesia compared to the sham control group within one week (F_1,8 _= 539, *P *< 0.001) (Figure 3A). By using a smaller microsurgical drill (0.5 mm diameter and 0.25 mm^2 ^area, equivalent to 25-gauge), we were able to induce slower development of pressure hyperalgesia as measured by the vocalization threshold in response to applied force upon stimulation of paravertebral tissues of the L4/L5 disk region compared to the sham control group (F_1,10 _= 19.8, *P *= 0.0012) (Figure 3B). Our microsurgical disk drill model demonstrated robust symptomatic discogenic back pain sustained for the behavioral pain test period (more than seven weeks after surgery). There was no secondary hyperalgesia as measured by the von Frey test for mechanical allodynia in the hind paws of both disk injury and sham control animals. Animals with disk drill puncture showed no pain response in the straight leg-raising test that is often used to diagnose patients with nerve compression-induced pain, such as that caused by herniated disks. The animals showed no change in performance using the rotarod test (data not shown).

### Disk degeneration-induced hyperalgesia is associated with disk narrowing

At the end of the nine weeks after initial surgery, the lumbar spinal motion segments were dissected and the gross disk heights were determined on the basis of X-ray imaging (Figures 2A to 2C). All harvested disks within a single rat were compared to each other and to the disks of all other rats. Significant disk space narrowing observed at the site of damaged disks was represented by approximately 55% reduced disk height compared to the sham control group (Figure 2C) (*P *< 0. 01). In an effort to rule out normal changes that occur with aging and/or altered motion as a result of disk injury that might affect neighboring disks, we compared the disks within a single animal (injured versus presurgery). Our results demonstrate that the disk heights of the presurgery group were similar to those of the sham control group (Figure 2A, naïve versus sham control). We did not observe significant changes in neighboring disks (L2/L3 and L3/L4) that might have been affected by the injured disks (data not shown). The disk height measurement was repeated twice at three-week intervals to validate the reproducibility of the disk height index. The intraobserver error of disk height index measurements was estimated to be minimal (within-group standard deviation (Sw) = 0.003 and coefficient of variation = 8.5%). The calculated interobserver error was Sw = 0.003 with a 9.5% coefficient of variation. The intraobserver reliability based on readings taken at two time intervals three months apart was κ = 0.98, showing excellent agreement.

### Disk puncture-induced hyperalgesia is associated with morphological changes in disks

Disks were harvested for histological assessments at nine weeks postsurgery. Morphological changes in disks of the sham control group (*n *= 6) and the 25-gauge needle puncture experimental group (*n *= 6) were compared in equivalent sections. We performed semiquantitative histological analyses using a grading system based on Safranin O and toluidine blue staining as described previously [19]. The control group displayed normal-looking disks with a Masuda grade of 4 (Figures 1A and 1B (left): Control), an intact AF with a normal pattern of fibrocartilage lamellas (U-shaped in the posterior aspect and slightly convex in the anterior aspect), a well-defined border between the AF and NP, and preserved proteoglycan content. In contrast, the experimental group (Figures 1A and 1B (right): Disk Injury) revealed severely damaged disks (Masuda grade 12) in which significant proteoglycan depletion was evident, most of the contents of the NP were lost and/or the NPs had collapsed (*P *< 0.05 by Mann-Whitney *U *test). At the nine-week time point, all the specimens had the maximum Masuda score of 12 points after disk injury. Importantly, at nine weeks, the vocalization threshold for force pressure was 410 ± 25 *g *in the disk puncture group versus 958 ± 26 *g *(*P *< 0.001), indicating a correlation between disk degeneration and sustained pain.

Interestingly, we observed that mast cells (visualized by intense toluidine blue staining), which are known to play a critical role in wound healing and inflammatory responses in connective tissue, were significantly increased at the site of injury in disks. We further validated our observation on the basis of immunohistochemical analyses by using an immune cell-specific antibody, CD11b. Our data demonstrate increased inflammatory responses at the site of disk injury as represented by highly increased immune reactivity with a CD11b antibody (Figure 1C: Control versus Disk Injury). Our observation of increased immuoreactivity to CD11b is consistent with all specimen sections with a maximum score of 12 points after disk injury.

### The pain pathways evoked by disk degeneration may overlap those of neuropathic pain

**Figure 4:**
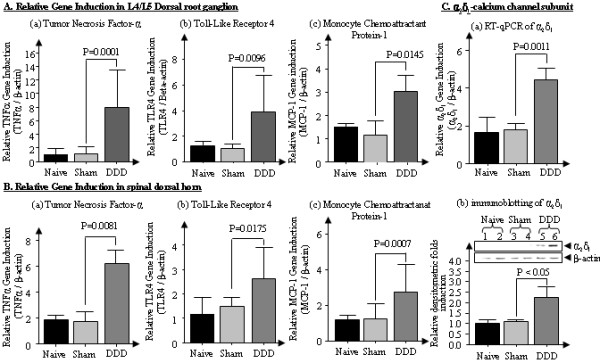
**Glial activity-associated gene analyses in rats with traumatic disk puncture-induced disc degeneration (DDD) by qRT-PCR and immunoblotting**. **(A) (a) **through **(c) **Dorsal root ganglion. **(B) (a) **through **(c) **Spinal dorsal horn. **(C) (a) **and **(b) **α_2_δ_1 _expression in spinal dorsal horn. Values are means ± SEM.

In neuropathic pain models, a set of pain modulators are upregulated (↑) or downregulated (↓) in sensory neurons in DRGs and spinal dorsal horn. These neuropathy molecules include CGRP(↓) [21], substance P(↓) [22] and BDNF(↑) [23], neuropeptide Y(↑) [24], galanin(↑) [25] via glial activation, which in turn activates the TNFα, MCP-1 and TLR4 pathways [26,27]. Similarly, we found highly stimulated expression of TNFα, MCP-1 and TLR4 in both the DRG (L4/L5) (Figures 4A(a) to 4A(c)) and the spinal dorsal horn (Figures 4B(a) to 4B(c)) in our animal model after disk degeneration with symptomatic behavioral chronic back pain. Furthermore, we observed significant increases in α_2_δ_1 _expression, a subunit of the voltage-gated calcium channel involved in neuropathic pain pathways, in the spinal dorsal horn of animals with discogenic pain on both the mRNA level (*P *< 0.0011) (Figure 4C(a)) and the protein level (*P *< 0.05) (Figure 4C(b)). These results suggest that the chronic pain pathways induced by disk degeneration may, at least in part, overlap with neuropathic pain. A comprehensive comparison of gene expression profiles between neuropathic pain and disk degeneration induced by NP removal is provided in Table 1[21-25,28,29].

**Table 1:** Altered gene expression in the sensory neurons in dorsal root ganglions in a neuropathic animal model compared to the traumatic disk injury-initiated chronic discogenic pain model

Tissues	Pain modulator	Neuropathic pain (sciatic nerve ligation)^a^, reference	Disk degeneration back pain model^b^, relative fold induction (*n *= 12) (*P *values)
Dorsal horn	Dynorphin	Upregulated [28]	1.941 ± 0.162 (*P *< 0.05)
	NK-1	Upregulated [29]	2.512 ± 0.521 (*P *< 0.05)
DRG	BDNF	Upregulated [23]	1.841 ± 0.312 (*P *< 0.05)
	NPY	Upregulated [24]	5.367 ± 0.474 (*P *< 0.05)
	CGRP	Downregulated [21]	0.487 ± 0.433 (*P *< 0.05)
	Substance P	Downregulated [22]	0.712 ± 0.251 (*P *< 0.05)
	Galanin	Upregulated [25]	1.1 ± 0.219 (equal)

BDNF = brain-derived neurotrophic factor; CGRP = calcitonin gene-related peptide; DRG = dorsal root ganglion; NK-1 = neurokinin 1; NPY = neuropeptide Y. ^a^Upregulated and downregulated expression patterns are relative to sham controls. ^b^25-gauge drilling was performed in 12 mice; data are means ± SEM.

### Pharmacological drug tests for therapeutic modulation of chronic back pain induced by disk degeneration

**Figure 5:**
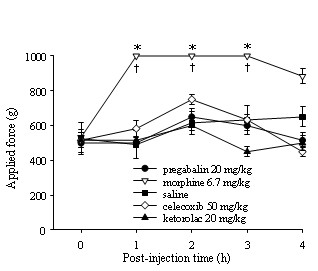
**Measurement of the vocalization threshold using a pressure algometer test to examine the pharmacological efficacy of drugs on traumatic disk puncture-initiated discogenic pain (tested during weeks 7 through 9)**. Administration of pregabalin (20 mg/kg), morphine (6.7 mg/kg), celecoxib (selective inhibitor of COX-2, 50 mg/kg), ketorolac (COX-1 and COX-2 inhibitor, 20 mg/kg) or control vehicle by either oral gavage or intraperitoneal injection followed by primary pressure pain assessment by algometer. Morphine demonstrated a potent analgesic drug effect with a single administration, whereas pregabalin, celecoxib and ketorolac use led to no significant behavioral changes compared to saline control. *P < 0.001 compared to the other drug groups; †*P *< 0.001 compared to presurgery baseline (time 0). COX-1 = cyclooxygenase 1; COX-2 = cyclooxygenase 2.

Therapeutic modulation of chronic pain using pharmaceutical drugs was investigated in the animal model. Because we observed that animals with sham abdominal incisions did not show any hyperalgesia (Figure 1), which suggests that their pain experience was not due to the abdominal incision, we selected rats with disk injury (but not sham animals) for testing with a panel of drugs, including morphine, celecoxib, ketorolac and pregabalin. We included morphine as a control prototype drug that is effective for certain types of pain (for example, nociceptive, inflammatory and postoperative pain). The potency of morphine in reducing primary pressure hyperalgesia may indicate a nociceptive process due to injury at the disks. Ketorolac and celecoxib are drugs used for inflammatory pain, but they are not effective for nociceptive pain (acute). Pregabalin is effective for neuropathic pain but not inflammatory or nociceptive pain. Pain behavior related to noxious stimuli was completely reversed by the administration of morphine sulfate (6.7 mg/kg) as assessed by vocalization threshold measurements using an algometer (F_4,10 _= 47.3, *P *< 0.001). The selective COX-2 inhibitor celecoxib (50 mg/kg), the nonsteroidal anti-inflammatory drug ketorolac (20 mg/kg) and the drug pregabalin (20 mg/kg) did not have significant antihyperalgesic effects (Figure 5).

## Discussion

The results of this study demonstrate the effects of puncture-induced intervertebral disk injury degeneration and pain behavior (pressure hyperalgesia) over a period of seven weeks in a rat model. The novelty of our animal model is that we were able to correlate structural and biochemical changes in intervertebral disk to disk degeneration-associated chronic back pain assessed by behavioral changes. In our animal model, the development of disk-related hyperalgesia was gradual and reached a maximum pain threshold four weeks after disk injury. Fast or slow development of pain symptoms may depend on the impact of the initial injury. The use of a larger drill (0.8 mm) resulted in more rapid development of behavioral changes compared to a smaller drill (0.5 mm). These behavioral changes may be comparable to those reported by other groups in which disk degeneration-associated pain was induced by the potent immune booster Complete Freund's Adjuvant (CFA). In the CFA-injected rats, rapid pain development was evident within one day after intradisk injection with CFA [30]. Immediate symptomatic pain caused by CFA-induced disk degeneration may be due to severe inflammatory response in the tissues, whereas disk puncture injury may generate much milder inflammation. More importantly, CFA-injected rats developed mechanical allodynia assessed by von Frey test in the hind paws, whereas no development of statistically significant secondary hyperalgesia (mechanical allodynia) was observed in animals with disk degeneration. It is important to note that clinically significant mechanical allodynia does not develop in patients with discogenic back pain (unpublished observations - Dr. Asokumar Buvanendran).

In the current study, we generated rat lumbar disk degeneration by puncturing two consecutive disks (L4/L5 and L5/L6). The consecutive disk degeneration procedure provides a model replicating the clinical reality that patients with symptomatic disk degeneration likely have multiple disk defects (albeit, presumably, with different degrees of disk degeneration). Our model facilitates pain assessments with greater sensitivity (more vocalization) than single-disk degeneration. Owing to the relatively small size of the animals (entire lumbar spinal column only 2 cm in length), we could not assess the degree of pain for individual lumbar disks. For example, it is not feasible to examine pain differences between the L3/L4 and L5/L6 disks. Our gene expression profiles suggest that chronic back pain may have a neuropathic component and may be linked to neuropathic pain pathways. The findings of this work corroborate the results of other studies of osteoarthritis-related chronic pain in symptomatic knee joints [19].

Our results obtained with pharmacological drug treatments suggest that the current animal model for disk degeneration-initiated pain recapitulates responses to human symptoms. For example, the potency of morphine (a positive control in the drug treatment in our study) in reducing primary pressure hyperalgesia may indicate a nociceptive process due to injury at the disks. The relative ineffectiveness of anti-inflammatory drugs in our animal model suggests that disk degeneration-induced chronic back pain might have resulted from a distinct pain pathway and might differ from other chronic pain conditions induced by an inflammatory pain source. Although oral administration of pregabalin did not attenuate pressure hyperalgesia in the disk degeneration model, even at the highest dose (20 mg/kg), our gene expression profiling suggests that disk degeneration-induced pain mechanisms may, at least in part, overlap with neuropathic pain mechanisms by significantly stimulating α_2_δ_1 _calcium subunits at the peripheral and central nervous system in both pain conditions.

In these studies, we performed a single analgesic administration for each drug, based on previously published studies by our group and others. For example, a single p.o. dose of pregabalin 10 mg/kg completely reversed mechanical allodynia in the spinal nerve ligation model (the Chung model [9]) for at least five hours [31], and a single 3 mg/kg dose of ketorolac completely abolished pain-related behavior in a postoperative pain model [15]. Importantly, the doses of celecoxib used in the current studies were higher than the half-maximal effective dose (ED_50_) for reversal of paw pressure hyperalgesia (ED_50 _= 4.1 mg/kg/dose) [32]. Thus these drugs may not be effective in alleviating discogenic pain pathway, but are effective blockers of other pain symptoms such as neuropathy or postoperative pain.

Although dosing in humans generally is less than 0.1 mg/kg, none of the drug concentrations used for the animals in this study caused sedation or motor incoordination. For example, administration of morphine (6.7 mg/kg) showed dramatic pharmacological antihyperalgesic effects without statistical differences in tests that monitor balance (the rotarod performance test) or spontaneous exploratory activity (for example, ambulation and rearing). These species differences in morphine drug responses may be due to distinctions in neurological parameters and pharmacokinetics. In one of our earlier studies with the most widely used postoperative pain model in rats (the Brennan model [18]), the ED_50 _needed to achieve antihyperalgesic effects was 3 mg/kg morphine, but 1 mg/kg morphine did not show any analgesic effects in rats [33].

It should be noted that although intervertebral disk degeneration is quite common and thought to be part of the aging process, the etiology and source of pain due to intervertebral disk degeneration is not well understood. The severity of disk degeneration does not always correlate with the degree of pain. Diagnosis of chronic pain due to disk degeneration is complex and based on multiple criteria, including clinical findings, as well as on radiographic and MRI and/or invasive discography. The animals with acute disk injury in our study exhibited increased behavioral hyperalgesia after surgery that persisted for the entire pain assessment period of seven weeks. Despite the strengths of our study, it remains unclear whether the animals would heal from lesser injuries. Furthermore, we have not been able to correlate the severity of pain with the degree of disk degeneration assessed by biochemical, histological and imaging analyses. Future time-course studies designed to correlate biochemical, histological and structural changes and pain scores at the early, intermediate and end stages of disk degeneration within and beyond seven weeks may be valuable for determination of the etiology of discogenic pain and pain sources by evaluating alterations in properties of peripheral disk tissues, disk heights, subchondral end plates and sensory nerve ingrowth, as well as changes in pain modulators in the nervous system.

## Conclusions

Our findings provide the basis for future research on new therapeutic interventions that may lead to improvements in the treatment of patients with back pain due to disk degeneration.

## Abbreviations

AF: annulus fibrosus; BDNF: brain-derived neurotrophic factor; CGRP: calcitonin gene-related peptide; COX-1: cyclooxygenase 1; COX-2: cyclooxygenase 2; DRG: dorsal root ganglion; i.p.: intraperitoneal; MCP-1: monocyte chemoattractant protein 1; MIA: monoiodoacetate; MRI: magnetic resonance imaging; NK-1: neurokinin 1; NP: nucleus pulposus; NPY: neuropeptide Y; p.o.: oral gavage; PCR: polymerase chain reaction; qRT-PCR: quantitative real-time-polymerase chain reaction; RT: reverse transcription; TLR: Toll-like receptor; TNF: tumor necrosis factor.

## Competing interests

The authors declare that they have no competing interests.

## Authors' contributions

JSKi, JSKr and HJI designed the study, generated both low-back pain animal models, performed the animal behavioral tests, generated the data, performed the data analysis and prepared the manuscript. AB designed the study, generated both low-back pain animal models, performed the data analysis and prepared the manuscript. HAS, KJT and DC designed the study, performed the data analysis and prepared the manuscript. XL and DY performed the animal behavioral tests, generated the data, performed the data analysis and prepared the manuscript. AJW performed the data analysis and prepared the manuscript. All authors read and approved the final version of the manuscript for publication.
